# The 10 largest public and philanthropic funders of health research in the world: what they fund and how they distribute their funds

**DOI:** 10.1186/s12961-015-0074-z

**Published:** 2016-02-18

**Authors:** Roderik F. Viergever, Thom C. C. Hendriks

**Affiliations:** Radboud University Medical Center, Radboud Institute for Health Sciences, Nijmegen, The Netherlands; Department of Metamedica, VUmc, Amsterdam, The Netherlands

**Keywords:** Globalization, Health funding, Health policy, Health research, Priority setting, Research and development (R&D), Research funding, Research governance, Research grants, Research policy

## Abstract

**Background:**

Little is known about who the main public and philanthropic funders of health research are globally, what they fund and how they decide what gets funded. This study aims to identify the 10 largest public and philanthropic health research funding organizations in the world, to report on what they fund, and on how they distribute their funds.

**Methods:**

The world’s key health research funding organizations were identified through a search strategy aimed at identifying different types of funding organizations. Organizations were ranked by their reported total annual health research expenditures. For the 10 largest funding organizations, data were collected on (1) funding amounts allocated towards 20 health areas, and (2) schemes employed for distributing funding (intramural/extramural, project/‘people’/organizational and targeted/untargeted funding). Data collection consisted of a review of reports and websites and interviews with representatives of funding organizations. Data collection was challenging; data were often not reported or reported using different classification systems.

**Results:**

Overall, 55 key health research funding organizations were identified. The 10 largest funding organizations together funded research for $37.1 billion, constituting 40% of all public and philanthropic health research spending globally. The largest funder was the United States National Institutes of Health ($26.1 billion), followed by the European Commission ($3.7 billion), and the United Kingdom Medical Research Council ($1.3 billion). The largest philanthropic funder was the Wellcome Trust ($909.1 million), the largest funder of health research through official development assistance was USAID ($186.4 million), and the largest multilateral funder was the World Health Organization ($135.0 million). Funding distribution mechanisms and funding patterns varied substantially between the 10 largest funders.

**Conclusions:**

There is a need for increased transparency about who the main funders of health research are globally, what they fund and how they decide on what gets funded, and for improving the evidence base for various funding models. Data on organizations’ funding patterns and funding distribution mechanisms are often not available, and when they are, they are reported using different classification systems. To start increasing transparency in health research funding, we have established www.healthresearchfunders.org that lists health research funding organizations worldwide and their health research expenditures.

**Electronic supplementary material:**

The online version of this article (doi:10.1186/s12961-015-0074-z) contains supplementary material, which is available to authorized users.

## Background

Approximately 40% of all health research in high-income countries is funded by public and philanthropic funding organizations [1]. These organizations play a central role in the development of new knowledge and products, particularly in areas that are not sufficiently profitable [2]. For example, the involvement of public and philanthropic funding organizations has been key in the development of new medical products to combat neglected diseases [1, 2] and, since recently, these organizations are increasingly taking action to address the lack of development of new antibiotics [3–5].

Transparency on who the main funding organizations of health research are, on what they fund (their funding patterns) and on how they decide on what gets funded (their priority setting mechanisms and funding distribution mechanisms) can help funding organizations to synchronize their efforts, potentially preventing the duplication of research and improving collaboration on research priorities, and has various other strategic and practical benefits for funders [2, 6–12]. Such transparency also allows for external evaluation of funding organizations’ portfolios and decision-making processes [7, 13]. This is particularly important for public funding organizations, since they distribute public funds. For philanthropic funders, such accountabilities are less clear, but given the substantial impact of some of these funders on the global landscape for health research, it might be reasonable to make similar demands from this group of funders [14, 15].

Although substantial insight has been created in recent years into countries’ expenditures on health research [1, 16–20], there has been relatively little scrutiny of the funding patterns and mechanisms of individual funding organizations. Mappings of individual funding organizations’ expenditures on health research are often limited to one or several countries [7, 10, 21–26] or to a select group of diseases [25, 27–29]. To increase the available information on major public and philanthropic funders of health research, we present a mapping in this article that had a simple target: to identify the 10 largest public and philanthropic funders of health research in the world, to report on what they fund, and on their mechanisms for distributing these funds (funding organizations’ priority setting mechanisms were beyond the scope of this study – see Limitations section for more detail).

## Methods

Here, we outline the methods used to identify the 10 largest funding organizations of health research in the world, and to assess the funding patterns and funding distribution mechanisms of these organizations. A more detailed description of these methods is provided in Additional file 1. All data were collected from November 4, 2013, to August 12, 2014.

### Identifying the 10 largest funders of health research

#### Search strategy

This study distinguished between four types of public and philanthropic health research funders: (1) public national or regional funders (excluding funders of official development assistance (ODA) and multilateral funders), (2) philanthropic funders, (3) ODA funders, and (4) multilateral funders. The mandate of the funding body did not need to be limited to funding health research. Funding organizations were identified through a search strategy that had several components: key funding organizations in the 20 countries with the highest spending on health research [1] were identified, membership lists of collaborative groups of funders (i.e. groups where major funders of health research collaborate on a global or regional level) were reviewed, publicly available lists of funding organizations that included annual spending on health research were searched, and data on Development Assistance for Health were used to identify key ODA funders. For every funder type, a specific search strategy was used to identify the largest funders of health research (Additional file 1). Private for-profit funding organizations were not included in our analysis; we only aimed to map public and philanthropic funders (private for-profit health research funders are mapped elsewhere [30]). Product development partnerships (PDPs) and other public private partnerships (PPPs) were also excluded because they are intermediate funding organizations, who are funded in turn by governments, philanthropies and the for-profit sector. Furthermore, we excluded single disease funders; although the majority of philanthropic funders of health research focuses on one disease [21], the largest philanthropic funders of health research tend to fund across multiple disease areas (with some exceptions [31, 32]). We note that the annual health research expenditures of the largest PDP, PPP and single-disease funders that we are aware of are lower than the annual expenditures of the 10 largest public and philanthropic funders reported in this study (see Additional file 1). Finally, in two cases (the United States Department of Defense (US DoD) and the European Commission (EC)) we included both the overarching organization at its largest sub-organizations or sub-programmes, because of the substantial differences between the funding distribution mechanisms of these sub-organizations and sub-programmes.

To aid future analyses of this kind, we provide an overview of various sources that helped us identify the main public and philanthropic funders of health research globally in Additional file 2.

#### Assessing health research expenditures

For all the funding organizations that followed from our search, publicly available data were collected on the organizations’ annual health research expenditures (from annual reports and websites). Data were collected for the most recent year available. When we were not able to find data on organizations’ annual expenditures in the public domain, we contacted funders to ask if they could provide us with their annual expenditures on health research.

Funding organizations differ on at least three aspects in terms of how they report their annual health research expenditures. First, expenditures can be reported as actual expenditures, commitments or budgets. Second, there can be differences in terms of what the expenditures cover. They can cover the organization’s total expenditures on health research excluding operational costs (for managing the funding organization), its total expenditures including operational costs, or its total overall turnover over a single fiscal year (this was only collected if the funding organization exclusively funded health research). Third, there can be differences in terms of the research areas that the reported expenditures pertain to: only health research, or broader categories such as health and biological research or life sciences research. For each funder we extracted data on annual health research expenditures in a step-wise manner, always reporting the actual expenditures excluding operational costs in the area of health research when possible. When these numbers were not available, we reported the next best available number, following the order in the categories provided above. We note that the data from the funding organizations in the top 10 all relate only to health research, all concern actual expenditures or commitments, and for all, except one, operational costs were excluded.

Training support and research education were not included in the overall amount for health research expenditures. In addition, for government ministries, we excluded two types of funding flows. First, when funding was provided by ministries to funding agencies for distribution, we included the funding for the funding agencies, but not for the ministries. Second, for government ministries, such as ministries of education or health, we excluded block funding to universities or hospitals (similar to other initiatives that have reported on health research funding flows [24]). For funding agencies, we did include institutional funding.

Finally, organizations’ expenditures were made comparable using methods by Young et al. [17, 20]. To do so, we first deflated organizations’ expenditures in the national currency to the year 2013 using Gross Domestic Product deflators from the International Monetary Fund World Economic Outlook Database of April 2014 [33]. Second, we converted the inflation-corrected expenditures to US dollars using the World Bank Official exchange rates for the year 2013. As a secondary outcome, we calculated funding organizations’ health research expenditures as 2013 purchasing power parity-adjusted US dollars (these are not reported in this article, but are available on www.healthresearchfunders.org) [17, 20].

### Assessing the funding patterns and funding distribution mechanisms of the 10 largest funders of health research

After the 10 largest funding organizations of health research were identified, data were collected on their funding patterns and funding distribution mechanisms. For each organization, data were collected on:

1. The funding mechanisms used to distribute funding: intramural funding or extramural funding. For extramural funding, we distinguished between project grants, ‘people grants’, programme grants, funding distributed to organizations and other extramural research funding. For project grants, data were collected to assess if the distribution was untargeted, targeted or highly targeted (for definitions see Additional file 1).
2. The amount of funding allocated to a list of 20 key health areas from the Global Burden of Disease classification [34].

Funding for operational expenditures was excluded.

Finally, we denoted whether funding organizations used a classification system to classify funding to various health areas and whether they reported statistics on funding for various research types (e.g. biomedical research, clinical research, epidemiological research or health systems research [35]) and recipient countries or regions.

All data were collected from online reporting databases, annual reports, official websites, or other information sources. After this, each funder was invited to participate in an interview. Before the interview, a document with collected data was made available to a representative of the funder. Before and during the interviews, representatives were asked to add, amend or confirm the data.

## Results

### Identifying the 10 largest funding organizations of health research

#### Public and philanthropic funding organizations

Our search identified 55 public and philanthropic funders that were candidates for being one of the 10 largest funders of health research in the world (Table 1), excluding ODA funders and multilaterals (we searched separately for these and report on them later). For 41 organizations, data on the organizations’ annual health research expenditures were available. For five of these organizations, this information was received through personal communications (not publicly reported). Fourteen funders did not provide figures about their annual health research expenditures. Often, these organizations were general funders of research and did provide overall expenditure data but not for health research specifically.

**Table 1:** Annual health research expenditures of 55 major public and philanthropic funders of health research

#	Public and philanthropic health research funding organizations	Country	Type of funding organization	Year for which funding data were collected	Total health research expenditures (in million 2013 US $)	Specificity of the funding data^a^	Research area that the funding data refer to^a^	Reporting format of the funding data^a^
1	National Institutes of Health (NIH)	USA	Public	2013	26,081.3	I	A	1
2	European Commission (EC)^b^	EU	Public	2013	3717.7	II	A	1
	*2a FP7 - Cooperation programme - Health Theme/Health Directorate* ^*c*^	EU	Public	2013	1181.7	II	A	1
	*2b European Research Council (ERC)*	EU	Public	2013	783.4	II	A	1
3	UK Medical Research Council (MRC)	GBR	Public	2013	1321.5	I	A	1
4	Institut national de la santé et de la recherche médicale (Inserm)	FRA	Public	2013	1041.2	I	A	1
5	United States Department of Defense (US DoD)^b^	USA	Public	2013	1017.7	I	A	2
	*5a Congressionally Directed Medical Research Program (CDMRP)*	USA	Public	2012	409.0	I	A	1
6	Wellcome Trust^d^	GBR	Philanthropic	2013	909.1	I	A	1
7	Canadian Institutes of Health Research (CIHR)	CAN	Public	2012	883.6	I	A	1
8	Australian National Health and Medical Research Council (NHMRC)	AUS	Public	2013	777.6	I	A	1
9	Howard Hughes Medical Institute (HHMI)	USA	Philanthropic	2013	752.0	I	A	1
10	Deutsche Forschungsgemeinschaft / German Research Foundation (DFG)	DEU	Public	2012	630.6	I	A	1
11	National Natural Science Foundation of China (NSFC)	CHN	Public	2012	621.3	II	A	1
12	Centre National de la Recherche Scientifique (CNRS)	FRA	Public	2013	531.0	III	B	2
13	UK Department of Health / National Institute for Health Research (NIHR)	GBR	Public	2012	491.2	I	A	1
14	Japan Society for Promotion of Science (JSPS)	JPN	Public	2011	472.5	I	A	1
15	Bundesministerium für Bildung und Forschung / Federal Ministry of Education and Research of Germany (BMBF)	DEU	Public	2013	472.1	I	A	1
16	Bill & Melinda Gates Foundation (BMGF)^e^	USA	Philanthropic	2011	462.6	I	A	1
17	Ministero della Salute / Ministry of Health of Italy	ITA	Public	2007	438.6	I	A	2
18	Instituto de Salud Carlos III (ISCIII)^e^	ESP	Public	2011	388.2	I	A	1
19	Ministry of Health of China	CHN	Public	2011	371.7	I	A	1
20	Japan Science and Technology Agency (JST)^e^	JPN	Public	2012	338.5	I	C	2
21	Institut Pasteur	FRA	Philanthropic	2013	220.9	I	A	1
22	Singapore National Medical Research Council (NMRC)	SGP	Public	2012	220.7	–	A	1
23	Korean National Research Foundation (NRF)	KOR	Public	2011	191.5	I	A	1
24	Consejo Nacional de Investigaciones Científicas y Técnicas (CONICET)^e^	ARG	Public	2012	184.4	II	B	1
25	Vetenskapsrådet-Medicine / Swedish Research Council	SWE	Public	2012	177.9	–	A	1
26	Swiss National Science Foundation (SNSF)	CHE	Public	2012	172.9	I	A	1
27	ZonMw / Netherlands Organisation for Health Research and Development	NLD	Public	2012	172.7	I	A	1
28	Sao Paulo Research Foundation (FAPESP)^e^	BRA	Public	2012	154.2	I	A	1
29	Indian Council of Medical Research (ICMR)	IND	Public	2011	140.3	I	A	3
30	Fund for Scientific Research - Flanders (FWO)	BEL	Public	2010	136.9	I	A	1
31	Korea National Institute of Health (KNIH)	KOR	Public	2013	120.0	III	A	2
32	Forskingsrådet / Research Council of Norway	NOR	Public	2012	113.5	III	A	2
33	Conselho Nacional de Desenvolvimento Científico e Tecnológico (CNPq)	BRA	Public	2013	110.8	I	A	2
34	Fonds zur Förderung der wissenschaftlichen Forschung / Austrian Science Fund (FWF)	AUT	Public	2012	99.5	I	C	1
35	South African Medical Research Council (SA MRC)	ZAF	Public	2012	63.2	I	A	3
36	Health Research Council of New Zealand	NZL	Public	2012	61.6	III	A	1
37	Danish Council for Independent Research / Medical Sciences	DNK	Public	2012	58.5	II	A	1
38	Russian Foundation for Basic Research (RFBR)	RUS	Public	2013	53.6	III	B	2
39	Danish Council for Strategic Research (two programmes: Individuals, Disease and Society & Health, Food and Welfare)	DNK	Public	2012	40.3	II	C	1
40	Consejo Nacional de Ciencia y Tecnología (CONACYT)	MEX	Public	2010	21.9	II	A	2
41	South African Department of Science and Technology (DST)	ZAF	Public	2012	13.5	I	B	1
42	Agencia Nacional de Promocion Cientifica y Technologica (Agenica - ANPCyT)	ARG	Public	–	No data	–	–	–
43	Biomedical Research Council of the Singapore Agency for Science, Technology and Research (BMRC of A*STAR)	SGP	Public	–	No data	–	–	–
44	Ministry of Science and Technology of China (MOST)	CHN	Public	–	No data	–	–	–
45	Indian Department of Biotechnology (DBT)	IND	Public	–	No data	–	–	–
46	Indian Department of Science and Technology (DST)	IND	Public	–	No data	–	–	–
47	King Abdulaziz City for Science and Technology (KACST)	SAU	Public	–	No data	–	–	–
48	Le Fonds de la Recherche Scientifique (FNRS)	BEL	Public	–	No data	–	–	–
49	Lipi Indonesian Research Council	IDN	Public	–	No data	–	–	–
50	Ministry of Healthcare of the Russian Federation	RUS	Public	–	No data	–	–	–
51	National Research Foundation South Africa (NRF SA)	ZAF	Public	–	No data	–	–	–
52	Oswaldo Cruz Foundation (Fiocruz)	BRA	Philanthropic	–	No data	–	–	–
53	Rockefeller foundation	USA	Philanthropic	–	No data	–	–	–
54	Tubitak / Scientific and Technological Research Council of Turkey	TUR	Public	–	No data	–	–	–
55	Turkish Academy of Sciences (TUBA)	TUR	Public	–	No data	–	–	–
	Total for the 10 largest funders of health research				37,132.2			
	Total for funders 11 to 41				7116.2			
	Total for all 41 funding organizations for which data were available				44,248.3			

Dashes (“–”) indicate that no information was available. Funders of Official Development Assistance (ODA) and multilaterals funders are excluded from this table and are reported separately. Data presented in the table are from the most recent year available at the time of data collection. Reported expenditures on health research can differ from what funders themselves report, because we excluded operational costs for managing the funding organization where possible, and because we excluded funding for research education

^a^Funders report differently on their expenditures. Preferably, we collected information on the actual expenditures of a funder in the area of health research, excluding funders’ operational costs. However, this information was not always available. Therefore, we describe here the type of data that we collected, in terms of how funding organizations report annual health research expenditures (i.e. I, actual expenditures; II, commitments; or III, budgets), in terms of the research areas that their reported numbers pertain to (i.e. A, only health research; B, health and biological research; or C, life sciences research), and in terms of what the expenditures cover (i.e. 1, total expenditures on health research excluding operational costs; 2, total expenditures on health research including also operational costs; or 3, total overall turnover for the funder over a single fiscal year)

^b^Two funders consisted of several sub-programmes with very different funding distribution mechanisms and patterns. (1) For the European Commission: Under the EC the FP7 was the largest research program in FY 2007–2013. The ERC and Cooperation programme - Health theme (the Health Directorate is the executive agency for the latter) are both programmes run under FP7. Under the FP7 there are several funding programmes: Ideas – ERC, Cooperation programme, People – Marie Curie, Capacity Program, CIP and Euratom. Due to this large number of funding programmes, and the differences between the funding programmes, we reviewed the two largest funding programmes: the Cooperation – Health theme and the Ideas – ERC programme. (2) For the US DoD: The defence health program holds 14 research programmes. We chose to review the largest programme, which was identified as the CDMRP

^c^The ERC was able to provide figures for its funding distribution mechanisms in the area of Life Sciences, not of health research. However, the website www.healthcompetence.eu provided figures of health research funding by the ERC. For consistency, figures for the Health theme of the FP7 Cooperation programme were extracted from the same website. There are slight deviations between these figures and the health research spending reported by FP7 Cooperation programme itself

^d^The annual expenditures for the Wellcome Trust are a slight overestimation for health research spending. Under Medical Humanities and engagement, various non-research grants are provided as well as other activities (e.g. running the Wellcome library), but these were not reported on separately, and are therefore included under ‘Research’

^e^Information was collected from official websites and annual reports. For these five organizations, information was not publicly available, and was acquired through personal communication with a representative of the organization

For the 10 largest funders, health research funding totalled to $ 37.1 billion, approximately 40% of all spending on health research globally by public and philanthropic sources [1]. The United States National Institutes of Health (NIH) contributed the largest part of this amount, with $ 26.1 billion in health research funding in 2013. The largest philanthropic funder was the Wellcome Trust ($ 909.1 million). The Wellcome Trust and the Howard Hughes Medical Institute (HHMI) were the only two philanthropic funders among the 10 largest funders of health research; the other eight organizations were public funding bodies. All 10 funders came from Northern America, Europe or Oceania. The largest Asian funding organization identified was the National Natural Science Foundation of China (NSFC) ($ 621.3 million), the largest funder from Latin America and the Caribbean was Consejo Nacional de Investigaciones Científicas y Técnicas (CONICET) from Argentina ($ 184.4 million), and the largest African funder was the South African Medical Research Council (SA MRC) ($ 63.2 million).

#### ODA-agencies and multilaterals

The expenditures of ODA-agencies and multilaterals on health research were substantially smaller than the expenditures of the largest public and philanthropic funding organizations (Tables 2 and 3). The largest funder of health research through ODA was USAID ($ 186.4 million) and the largest multilateral funder was WHO ($ 135.0 million).

**Table 2:** Annual health research expenditures of key funders of Official Development Assistance (ODA)

Funding organization	Country	Year for which funding data were collected	Expenditures on health research (in million 2013 US $)	Specificity of the funding data^a^	Research area that the funding data refer to^a^	Reporting format of the funding data^a^
United States Agency for International Development (USAID)	USA	2012	186.4	II	A	1
UK Department for International Development (DFID)	GBR	2014	97.5	III	A	2
Grand Challenges Canada	CAN	2013	46.3	I	A	1
Dutch Directorate General of Development Cooperation (DGIS)	NLD	2012	11.7	I	A	1
German Federal Ministry for Economic Cooperation and Development (BMZ)	DEU	2011	0.9	I	A	1
Canadian Department of Foreign Affairs, Trade and Development (DFATD)	CAN	2011	0.8	I	A	1
Ministère des Affaires Etrangères et Européennes (MAEE)	FRA	2011	0.7	I	A	1
L'Agence Française de Développement (AFD)	FRA	2013	0.0	–	–	–

For DFID, USAID and Grand Challenges Canada data on annual health research expenditures were publicly available. For AFD data were acquired through a personal communication. For all other organizations, no data were available or could be provided. For these organizations, annual health research expenditures were approximated by extracting expenditure figures from G-FINDER, which is limited to health research focused on product development [54]

^a^See note ^a^ under Table 1

**Table 3:** Annual health research expenditures of key multilateral funding organizations of health research

Funding organization	Year for which funding data were collected	Expenditures on health research (in million 2013 US $)	Specificity of the funding data^a^	Research area that the funding data refer to^a^	Reporting format of the funding data^a^
World Health Organization (WHO)	2006	135.0	I	A	1
World Bank	2011	2.1	I	A	1
Pan American Health Organization (PAHO)	2011	0.0	I	A	1
Global Alliance for Vaccines and Immunisation (GAVI) (including the International Finance Facility for Immunisation (IFFIm))	2013	0.0	I	A	1
Global Fund to Fight AIDS, Tuberculosis and Malaria	2013	0.0	–	–	–
UNAIDS	2013	0.0	–	–	–
UNITAID	–	No data	–	–	–

WHO data on annual health research expenditures were derived from an article by Terry et al. [55]. The Global Fund and UNAIDS data were acquired through personal communications. For the other organizations, no data were available or could be provided. For these organizations, annual health research expenditures were approximated by extracting expenditure figures from G-FINDER, which is limited to health research focused on product development (not available for UNITAID) [54]. It should be noted that some of these organizations, such as the World Bank, conduct a substantial amount of health policy research every year. Since G-FINDER’s data were the only data available on World Bank expenditures on health research, policy research expenditures have not been included

^a^See note ^a^ under Table 1

### Assessing the funding patterns and funding distribution mechanisms of the 10 largest funding organizations of health research

#### Funding mechanisms used to distribute funding

There was considerable diversity in organizations’ funding distribution mechanisms (Table 4). Five funders funded research fully extramurally, five allocated at least a proportion of their funding to intramural research institutes, and one funder, the Institut national de la santé et de la recherche médicale (Inserm), funded research (almost) exclusively intramurally (total is 11 because for the EC and the US DoD we analysed the sub-organizations or sub-programmes: the US Congressionally Directed Medical Research Program (CDMRP), the Health theme of the EC FP7 Cooperation programme and the European Research Council (ERC)).

**Table 4:** Overview of funding distribution mechanisms of the 10 largest funding organizations of health research globally (in million 2013 US $)

	1	2	*2a*	*2b*	3	4	5	*5a*	6	7	8	9	10
Funding organization	NIH	EC – total	*EC – FP7 health*	*EC – ERC*	MRC	Inserm^b^	US DoD – total	*US DoD – CDMRP*	Wellcome Trust^a^	CIHR	NHMRC	HHMI	DFG
Country	USA	EU	EU	EU	GBR	FRA	USA	USA	GBR	CAN	AUS	USA	DEU
Total health research funding	26,081.3	3717.7	1181.7	783.4	1321.5	1041.2	1017.7	409.0	909.1	883.6	777.6	752.0	630.6
Year	2013	2013	2013	2013	2013	2013	2013	2012	2013	2012	2013	2013	2012
Intramural vs. extramural	Largely extramural	NA	Extramural	Extramural	Mixed	Intramural	NA	Largely extramural	Largely extramural	Extramural	Extramural	Largely extramural^c^	Extramural
Mechanism for extramural funding	Largely untargeted (but earmarked for broad areas), with smaller targeted and organizational funding streams	NA	Targeted, issuing calls under prioritized health research topics	Untargeted	Mixed approach, with large streams for untargeted (but earmarked for broad areas), targeted and organizational funding^d^	–	NA	Largely targeted, issuing calls under prioritized health research topics, with a smaller untargeted funding stream	Largely untargeted, with a smaller targeted funding stream	Largely untargeted, with a smaller targeted funding stream	Largely untargeted, with smaller organizational and targeted funding streams	Untargeted	Mixed approach, with large streams for organizational and untargeted funding and a smaller targeted funding stream
**Intramural funding**	3283	0	0	0	513	1041	–	16	155	0	0	100	0
**Extramural funding**	22,799	3718	1182	783	809	0	–	393	754	884	778	652	631^e^
Project grants	18,341	–	1182	783	519	0	–	368	–	692	478	0	271
* Untargeted*	0	–	0	783	0	0	–	0	–	464	468	0	217
* Untargeted, earmarked for broad areas*	11,708	–	0	0	220^f^	0	–	0	–	0	0	0	0
* Targeted*	3738	–	1182	0	221^f^	0	–	299	–	228	0	0	52
* Highly targeted*	2895	–	0	0	0	0	–	69	–	0	10	0	2
‘People grants’	615	–	0	0	98	0	–	25	–	105	156	652	14
All funding to organizations	2709	–	0	0	163	0	–	0	–	4	143	0	254
Other research^g^	1134	–	0	0	28	0	–	0	–	83	0	0	1
Research training/science education^h^	768	–	0	0	0	0	–	26	–	64	0	80	38

All data are from publicly available documents, except for CDMRP and DFG, for which data were received through personal communication, and for CIHR for which the data were largely publicly available but were supplemented through personal communication

Zero’s (“0”) indicates an amount of zero million in funding; dashes (“–”) indicate that no information on the amount of funding was available; NA indicates that we did not extract information for overarching funding organizations (only for the more specific organization(s) next to it). Some of the funders we identified (the EC and the US DoD) consisted of several sub-organizations that distributed the research funding in different ways, in which case we compiled total funding for the organization as a whole, but analysed the distribution mechanisms of the largest sub-organization(s)

NIH, National Institutes Of Health; EC, European Commission; FP7 health, FP7-cooperation programme – Health Theme; ERC, European Research Council; US DoD, United States Department Of Defense; CDMRP, Congressionally Directed Medical Research Program; MRC, Medical Research Council; Inserm, Institut national de la santé et de la recherche médicale; CIHR, Canadian Institutes Of Health Research; NHMRC, National Health and Medical Research Council; HHMI, Howard Hughes Medical Institute; DFG, Deutsche Forschungsgemeinschaft

^a^The annual expenditures for the Wellcome Trust are a slight overestimation for health research spending. Under medical humanities and engagement, various non-research grants are provided as well as other activities (e.g. running the Wellcome library), but these were not reported on separately, so are included under ‘Research’

^b^Inserm funds research almost exclusively intramurally. However, Inserm does provide for some extramural funding. We were not able to find any information about the proportion of funding by Inserm that is distributed extramurally, but it is likely very small, so we classified all of Inserm’s funding as intramural. Historical research into Inserm’s expenditures put the organization’s intramural/extramural proportions of distributed funds at 10/90 [23]

^c^Technically, HHMI funds research 100% intramurally because it employs its own researchers. However, its researchers are mostly located at external research institutes or universities (with the exception of HHMI’s Janelia Research Campus), and therefore, for this table, we classified HHMI’s funding distribution mechanism as largely extramural. The 100 million dollar for intramural funding is based on an annual budget estimate from 2010 for the Janelia Research Campus by HHMI itself and is not a precise number

^d^The MRC has both untargeted and targeted funding streams; most are untargeted (‘response-mode’ in the MRC’s description). However, untargeted funding at the MRC is not fully untargeted, the Research Boards that make funding decisions take into account broader strategic considerations, priorities, portfolio balance, and issue ‘highlight notices’ for issues that are of especial importance to the MRC

^e^The data for DFG do not add up because multiple sources were used (public reporting and personal communication). The total amount of funding includes overhead costs for projects, while amount for various research categories are exclusive of overhead costs

^f^Funding distribution figures of MRC are based on data provided in the annual report 2013/2014 of the MRC United Kingdom. All figures are based on actual expenditures; however, targeted and untargeted funding are based on commitments made, and therefore do not add up to total Project funding

^g^‘Other research’ included ‘other research’ categories in funding organizations’ reports and funds for research communication when specified separately

^h^Training support and research education were not included in the overall amount for health research expenditures, but because there can be an overlap between these activities and research activities, particularly in the case of post-doctoral fellowships, we collected expenditures toward research education and training separately

For definitions of terms in this table (e.g. ‘people grants’ or ‘untargeted’/‘targeted’/‘highly targeted’) see Additional file 1

Of the 10 funding organizations that provided extramural funding, for six, the main mechanism for extramural funding distribution was the allocation of funding through untargeted competitive project or investigator grants (often, there were also some smaller programmes that used a more targeted distribution). Two funders, the Health theme of the European Commission FP7 Cooperation programme and the US CDMRP, used a more targeted approach and issued calls under prioritized areas. Funders also made use, in varying degrees, of highly targeted funding schemes, such as research contracts, tenders or prizes, but this was never the dominant form of funding distribution. The last two funders, the United Kingdom Medical Research Council (MRC) and the Deutsche Forschungsgemeinschaft (DFG), used a mixed approach to allocate funding, with substantial contributions made through different funding distribution mechanisms. Lastly, the funding model of the NIH and the untargeted part of the MRC deserve separate mentioning because, although they adhered largely to an untargeted model and research funding was available for all areas of health research, the amounts of funding available for various broad research areas were earmarked (in the case of the NIH, for example, through budgets for the NIH institutes). This differs from targeted approaches, where not all areas have to be funded and the prioritization is often more specific, but it is also not completely untargeted.

Finally, most funders mainly dispensed funding via project grants, with smaller programmes that provide grants to excellent individual researchers. However, others put more focus on individual excellence. The HHMI has traditionally been a proponent of such people-focused funding. Since recently, other funders, such as the Wellcome Trust and the NIH, are increasingly making use of ‘people grants’ as well [36].

#### Funding patterns towards diseases

The funding organizations’ research expenditures towards 20 specific health areas are shown in Table 5. We could report data for at least one health area for seven funders. However, as the table makes clear, these data were often not available.

**Table 5:** Overview of funding provided by the 10 largest funders of health research globally to 20 selected health areas (in million 2013 US $)

	1	2	*2a*	*2b*	3	4	5	*5a*	6	7	8	9	10
Funding organization	NIH	EC – total	*EC – FP7 health*	*EC – ERC*	MRC	Inserm	US DoD – total	*US DoD – CDMRP*	Wellcome Trust	CIHR	NHMRC	HHMI	DFG
Country	USA	EU	EU	EU	GBR	FRA	USA	USA	GBR	CAN	AUS	USA	DEU
Year	2013	–	2013	–	2009/2010	–	–	2012	2009/2010	2012	2013	–	–
**Communicable, maternal, neonatal, and nutritional disorders**	–	–	–	–	–	–	–	–	–	–	179	–	–
Infectious diseases	4887	–	186	–	168	–	–	–	171	242	127	–	–
* Lower respiratory infections*	113	–	–	–	–	–	–	–	–	–	14	–	–
* Diarrheal diseases*	–	–	–	–	–	–	–	–	–	–	8	–	–
* HIV/AIDS*	2898	–	–	–	–	–	–	–	–	46	12	–	–
Maternal disorders	–	–	–	–	30	–	–	–	14	–	32	–	–
* Maternal haemorrhage*	–	–	–	–	–	–	–	–	–	–	–	–	–
Neonatal disorders	486	–	–	–	–	–	–	–	–	–	19	–	–
* Pre-term birth complications*	198	–	–	–	–	–	–	–	–	–	15	–	–
Nutritional deficiencies	–	–	–	–	–	–	–	–	–	–	2	–	–
* Protein deficiencies*	–	–	–	–	–	–	–	–	–	–	–	–	–
**Non-communicable diseases**	–	–	–	–	–	–	–	–	–	–	–	–	–
Cardiovascular and circulatory diseases	1964	–	82	–	50	–	–	13	28	40	97	–	–
* Ischemic heart disease*	404	–	–	–	–	–	–	–	–	–	30	–	–
Neoplasms	5274	–	80	–	84	–	–	–	12	–	139	–	–
* Trachea, bronchus, lung cancer*	208	–	–	–	–	–	–	–	–	–	6	–	–
Mental health	2174	–	–	–	59	–	–	–	29	55	59	–	–
* Unipolar depressive disorder*	415	–	–	–	–	–	–	–	–	–	22	–	–
**Injuries**	367	–	–	–	1	–	–	–	0	–	35	–	–
Transport injury	–	–	–	–	–	–	–	–	–	–	2	–	–

Zero’s (“0”) indicates an amount of zero million in funding; dashes (“–”) indicate that no information on the amount of funding was available. Health areas were chosen to be a representative sample of health areas in the Global Burden of Disease (GBD) classification [34]. Adding spending on the various health areas per funder will not total to the funder’s total expenditures, because the categories are a selection of health areas from the GBD report. Funding for selected health areas needs to be interpreted with caution because these data are mutually exclusive for some funders (Wellcome, MRC, CIHR, EC FP7 health, CDMRP), but not for others (NIH, NHMRC)

See Table 4 for funders’ abbreviations

Sources: NIH, http://report.nih.gov/categorical_spending.aspx; EC FP7 Cooperation Progamme Health theme, http://www.healthcompetence.eu/; MRC and Wellcome Trust taken from the United Kingdom Health Research Analysis 2009/10 (United Kingdom Clinical Research Collaboration, 2012) (http://www.ukcrc.org/research-coordination/health-research-analysis/uk-healthresearch-analysis/3 and http://www.hrcsonline.net/pages/data); CDMRP, funding categorisation based on “physiology classification system” that we were provided by CDMRP through personal communication; CIHR, data received through personal communication; NHMRC, http://www.nhmrc.gov.au/grants/research-funding-statistics-and-data/funding-datasets . Funding for selected health problems was only reported if funders categorized grants using an indexing system, amounts for various targeted programmes were not included, with the exception of the EC FP7 Cooperation programme, health theme, who requested we use programme-based figures for annual commitments from HealthCompetence.eu

Funding patterns varied, with some funders showing preferences for investing in non-communicable over communicable diseases and others showing the opposite. For example, the NIH spent less on infectious disease research in total than on cancer research alone, while the Wellcome Trust spent 14 times more on infectious disease research than on cancer research. Similar variations arose when comparing more specific disease areas within the non-communicable or communicable diseases. For example, the NIH spent almost three times more on cancer research than on cardiovascular research while the EC under the FP7 programme spent roughly equal amounts on both, and while HIV/AIDS funding comprised more than half of the infectious disease research funding at the US NIH, it comprised less than 10% of that funding at the Australian National Health and Medical Research Council (NHMRC).

Six funders used classification systems to classify their funding to health areas (Table 6); five different classification systems were used by these funders (the two funders from the United Kingdom used the same system). Besides using different categories for health problems, these systems also varied on other aspects, such as who enters the data (e.g. the researcher or a specialist employed by the funder) and whether grants can be indexed as belonging to one or multiple health problems. Seven funders reported amounts of funding allocated to various research types and the same seven reported how much funding was allocated to various recipient countries or regions.

**Table 6:** Funding organizations’ use of classification systems for reporting health research expenditures

	Funding organization	Country	Health categories	Recipient countries or regions	Type of research	Data classification system for health categories
1	NIH	USA	Yes	Yes	Yes	RCDC
2	EC	EU	No	No	No	–
*2a*	*FP7 Cooperation programme, health theme*	EU	No	Yes	Yes	–
*2b*	*ERC*	EU	No	No	No	–
3	MRC	GBR	Yes	Yes	Yes	HRCS (modified ICD-10)
4	INSERM	FRA	No	–	–	–
5	US DoD	USA	No	–	–	–
*5a*	*CDMRP*	USA	Yes	Yes	Yes	"Physiology" classification^b^
6	Wellcome Trust	GBR	Yes	Yes	Yes	HRCS (modified ICD-10)
7	CIHR	CAN	Yes^a^	Yes	Yes	EIS Research Area and Research Class taxonomy in Research Reporting system^a^
8	NHMRC	AUS	Yes	Yes	Yes	ANZSRC (modified ICD-10)
9	HHMI	USA	No	–	–	–
10	DFG	DEU	No	No	No	–

“–” means that we were not able to find information for this field

See Table 4 for funders’ abbreviations. ANZSRC, Australian and New Zealand Standard Research Classification; EIS, Electronic Information System; HRCS, Health Research Classification System; ICD, International Classification of Diseases; RCDC, Research, Condition, and Disease Categorization

^a^At the time of data collection, CIHR was in the process of implementing its data classification system. It did not publicly report data yet on how much funding goes to various health areas, but noted that it would do so in the future. CIHR was able to provide us with data for several health areas via personal communication. The organization noted through the same personal communication that, in terms of classifying or reporting on the research that it funds, CIHR uses the EIS Research Area and Research Class in-house menu, Institute in-house Keyword lists, the Thompson Reuters Fields and Sub-Fields, and MESH. The Research Reporting system uses the EIS Research Area and Research Class taxonomy (http://www.cihr-irsc.gc.ca/e/45318.html)

^b^This classification was sent to us by CDMRP upon request. All other classifications are publicly available

## Discussion

In this article, we have identified the 10 largest funding organizations of health research globally and shed more light on their funding distribution mechanisms and funding patterns. Two main conclusions can be drawn from this mapping of influential funders of health research.

### Differences between funding organizations: the need for more evaluation of funding distribution models

First, there is considerable diversity between funding organizations in terms of what they fund and how they distribute those funds. This begs the question: do some funding distribution models have more impact than others? The impact of different approaches to funding health research is regularly discussed in the literature, for example, for intramural versus extramural funding [23], for targeted versus untargeted funding [37], for ‘people grants’ versus project grants [36, 38], for small grants versus large grants [10], and for competitive versus non-competitive research funding [39]. However, comparative evaluations of the impact of various funding models are scarce [10, 23, 38], even though approaches to measure the impact of health research are available [40]. An exception has been the recent comparisons of ‘people grants’ versus projects grants in the United States, which compared HHMI with NIH researchers and NIH Pioneer Awards with NIH project grants [36, 41–43]. These comparisons have led the NIH to consider a broad shift toward ‘people grants’, demonstrating the value and potential impact of such evaluations [36]. Evaluations of this kind provide new insights when comparing funding models across funding organizations, but given the different contexts in which funders operate, comparing the impact of different models within one funding organization is perhaps particularly valuable and should become more common practice.

There is also a need for more debate about where the power to decide priorities for publicly funded health research should lie (with parliaments, ministries, funding agencies, or independent committees of experts). Such debate is needed because there are finite resources for investing in health research and thus priorities need to be set using fair and legitimate methods and using the best possible evidence [44]. In practice, public sector health research funding decisions are not only made on the basis of what research is needed, but are regularly influenced by other factors, such as political interests, advocacy and lobbying [2]. Thus, there is a need for transparency on who makes those decisions and to debate who should make them [2, 13, 45–47]. Analysis of funding organizations’ priority setting processes was not part of this study (see Limitations) but deserves to be a more frequent subject of research studies in the future.

### Improving publicly available data on health research funding

Second, to enable evaluation and debates as noted above, it is necessary to have a map of the health research funding landscape: to know who the main funders of health research are, what they fund, and how they decide what gets funded [2, 6–11, 13]. Yet, this study shows that these data are often not available. Through our study, we did not find a list of all public or philanthropic health research funders worldwide that included their annual health research expenditures (Additional file 1). Therefore, we have now established such a list ourselves at www.healthresearchfunders.org. On this website, we provide access to the data collected for this article and to information on more than 200 other public and philanthropic funders of health research that we have added to this website since the mapping for this article was completed.

Besides the absence of a global listing of funding organizations, we found that data on organizations’ funding patterns and funding distribution mechanisms are often not available, and when they are, they are difficult to aggregate, owing to differences in funders’ data classification systems. Notably, we only collected these data for the 10 largest funding organizations of health research. The absence of such information, and the difficulties in aggregating the data across funders, are likely to be more prominent when smaller funders are also included. There is currently no consensus on a framework for producing descriptive data on funders’ funding patterns (both in terms of health areas and research types) nor on a framework for describing their funding distribution mechanisms [6, 8, 37]. In this article, we have proposed three frameworks for reporting data on health research funding: for reporting data on funding distribution mechanisms (Table 4), for reporting data on funding patterns in terms of health problems (the Global Burden of Disease classification [34]), and for reporting data on funding patterns in terms of research types (biomedical research, clinical research, epidemiological research or health systems research, as proposed by Frenk [35]). The adoption of standards for reporting funding data, including guidance on what data classification systems to use, by funding organizations, for example through collaborative initiatives such as the Heads of International Research Organizations, would substantially improve the quality and comparability of reported funding data [9].

Funding organizations are starting to support the goal of transparency and are increasingly recognizing the problems noted above and addressing them. At the 2014 World Health Summit in Berlin, several major funders of health research expressed interest to work together toward developing a common approach for mapping health research funding flows [12]. Another good example of a multi-funder collaboration to increase insight in health research investments is the World RePORT website [48]. On a national level, the United Kingdom has led the way in terms of harmonized reporting by showing it is feasible to collect comparable data on health research funding from all major public funding bodies and charities in a country [22]. Besides initiatives from funders themselves, there are also several promising initiatives from other parties to address the lack of data on global health research funding [1, 16, 49–51]. The recent decision to establish a Global Observatory on Health R&D at WHO in particular may help to improve transparency in this area [1].

### Limitations

Finally, we note that the mapping conducted for this article has had several limitations. First, we have excluded funding organizations in the private for-profit sector (these are listed elsewhere [30]). Second, national systems for funding health research vary. In many countries, a large amount of health funding is dispersed directly from governments to universities or research institutes via block grants. We excluded these block grants and therefore the public funding organizations that we report on do not all contribute the same share of all health research that is publicly funded in a country. Third, we had to make several generalizations in order to be able to report data across funders that were diverse in their funding distribution mechanisms and reporting systems. For instance, what we have termed ‘targeted’ research funding, is a grey area that ranges from broad prioritized research areas to specific research topics or questions [52]. Also, funders reported on their expenditures on health research in various formats. Although we have kept track of these varying reporting formats, they decrease comparability across funders. Fourth, we would have liked to exclude overhead costs within project funding (not operational costs of the funder, which we did exclude where possible, but overhead costs of the research organization), to measure only the amount of funding that went to research, but this was not feasible because it was mostly not reported. Fifth, our proposed framework for reporting on funders’ funding distribution mechanisms (Table 4) lacks detail. It would have been interesting to also report on more detailed mechanisms, such as funders’ grants for businesses and PDPs/PPPs, but we did not include such analyses because of a lack of comparable data across funders. Sixth, funding organizations frequently make adaptations to their funding strategies, and therefore our findings should be viewed as a snapshot of funders’ expenditures, funding distribution mechanisms and funding patterns during the time of our data collection [53]. Seventh, in addition to reporting about funding organizations’ funding distribution mechanisms and patterns, we would have liked to report on funding organizations’ priority setting processes as part of this work (another important aspect of how funders decide what gets funded). However, we found that priority setting processes were generally not well-described and highly variable across funders, making it difficult to analyse and report our data. It deserves recommendation that such an analysis is conducted in the future, but the development of a framework for assessing priority setting processes at funders is needed first, potentially based on existing guidance for health research priority setting [44]. Lastly, and most importantly, our search strategy was limited in scope (see for more detail Additional file 1), was aimed only at finding the 10 largest funding organizations of health research in the world, and detailed data were only collected for those 10 organizations.

## Conclusions

This study identified the 10 largest funding organizations of health research in the world and showed that these organizations together fund research for $37.1 billion, 40% of all public and philanthropic health research spending globally. It also mapped the funding patterns and funding distributions mechanisms of these funders and showed that there is considerable diversity between organizations in terms of what they fund and how they distribute those funds, highlighting the need for comparative evaluations of the impact of different funding distribution models. Moreover, because many of the data we tried to collect were not available, our study demonstrates that there is a need for increased transparency on who the largest funding organizations of health research are, what they fund, and how they decide what gets funded. As a first step in improving transparency in this area, we have proposed frameworks for reporting on funding patterns (in terms of health problems and research types) and for reporting on funding distribution mechanisms in this article and have established www.healthresearchfunders.org, where we list more than 250 public and philanthropic funders of health research and their annual health research expenditures. We will further expand and update this list of funding organizations in the future and welcome both suggestions and data from all who wish to help us make this database more accurate and more inclusive.

## Additional files

Additional file 1:
**More detailed description of methods.** (DOCX 46 kb) Additional file 2:
**Sources for identification of health research funding organizations.** (DOCX 45 kb)
