# Supplementary material for: The 10 largest public and philanthropic funders of health research in the world: what they fund and how they distribute their funds
Source: Health Res Policy Syst. 2016 Feb 18;14:12. doi: 10.1186/s12961-015-0074-z (PMC4759950; doi:10.1186/s12961-015-0074-z)
Supplement: Additional file 1: — More detailed description of methods. (DOCX 46 kb) [file 12961_2015_74_MOESM1_ESM.docx]

# Additional File 2. More detailed description of methods

Here below, we provide a description of the methods used to identify the ten largest funding organizations of health research in the world, and to assess the funding patterns and mechanisms of these funders. This description adds to the Methods in the main text of the article by providing more detail.

## Identifying the ten largest funders of health research

This study distinguished between four types of health funders: 1) public national or regional funders of health research (excluding funders of official development assistance (ODA) and multilateral funders), 2) philanthropic funders of health research, 3) ODA funders, and 4) multilateral funders. The mandate of the funding body did not need to be limited to funding health research. For every funder type a specific search strategy was developed to identify the ten largest public and philanthropic funders of health research overall.

### Identification of major public funders of health research

Major public funders of health research (excluding ODA funders and multilateral funders) were identified by: 1) we reviewed the members of three collaborative groups of funders (i.e. groups where major funders of health research collaborate on a global or regional level), i.e. the Heads of International Research Organizations (HIROs), the African Network for Drugs and Diagnostics Innovation (ANDI), and Enhancing Support for Strengthening the Effectiveness of National Capacity Efforts (ESSENCE) for health research; 2) we identified the major national funders of health research in the G20 countries, in the 20 countries with the highest overall spending on health research, and in the 20 countries with the highest public spending on health research [1] (included countries: Argentina, Australia, Belgium, Brazil, Canada, China, Denmark, European Union, France, Germany, India, Indonesia, Italy, Japan, Mexico, Netherlands, Republic of Korea, Russia, Saudi Arabia, South Africa, Spain, Sweden, Switzerland, Turkey, United Kingdom, United States) by reviewing policy reports, grey literature and some published articles; and 3) we looked for publicly available lists of public funders that included annual spending on health research for each funder. We limited the amount of public funders that we included per country to two, to limit the scope of our study. The second largest public funder for each country was only included if it funded research for more than 200 million USD annually, or if it was significant in size as compared to other funding organizations in the country or to our compiled list of the largest funders in the world. This limitation was implemented to keep data collection feasible. We were specifically looking to identify the 10 largest funders of health research in the world for this study and felt this limitation was a reasonable balance between feasibility and inclusiveness. We checked for larger countries, particularly the United States, whether there were other funders besides the top-2, to make sure that we did not miss any funding organizations that would have ended up in our global top-10. These organizations are not listed in this article (for consistency of reporting) but are listed on [www.healthresearchfunders.org](http://www.healthresearchfunders.org) (the third largest public funder from the US was the U.S. Department of Veterans Affairs ($ 582.0 million)).

Countries’ health research systems vary substantially. As a result, it was often not easy to identify the primary public funder(s) of health research. In addition, for several countries, very little information was publicly available about the health research system. Where we could, we used reports on countries’ health research systems to identify the primary public funder(s) of health research (e.g. [2–15]).

### Identification of major philanthropic health research

Major philanthropic funders of health research were identified by: 1) we reviewed the members of three collaborative groups of funders as for public funders; and 2) we looked for publicly available lists of philanthropic funders that included annual spending on health research for each funder.

### Identification of major ODA-agencies that fund health research

ODA-agencies with major spending on health research were identified by: 1) The world’s top-5 donor countries of Development Assistance for Health (DAH) were identified.[16] For each of these countries, the major ODA agencies were identified and their expenditures on health research were assessed. 2) The annual health research expenditures of the three largest ODA-funders of health research in the area of neglected diseases were extracted from a recent report on global funding for neglected diseases.[17]

### Identification of major multilateral funders of health research

Major multilateral funding organizations of health research were identified by: 1) First, the world’s top-3 donor countries of DAH were identified.[16] Second, multilaterals that received investments from these three donor countries were identified. Third, the annual health research expenditures of these multilaterals were assessed. 2) The annual health research expenditures of the three largest multilateral funders of health research in the area of neglected diseases were extracted from a recent report on global funding for neglected diseases.[17]

### Overarching and sub-organizations

In two cases, we identified an overarching funding organization that consisted of sub-organizations or -programmes that funded research using distinctly different funding mechanisms (the European Commission (EC) and the United States Department of Defense (US DoD). In these two cases, we included both the overarching funding organization and its major sub-organizations or -programmes. For the US DoD, we also included the Congressionally Directed Medical Research Program (CDMRP). The EC is the organization responsible for the Framework Program 7 (FP7), ending the 31^st^ of December 2013, and the Horizon2020 program, starting the 1^st^ of January 2014 (we evaluated numbers for the FP7 program). For the EC, we also included the FP7’s two largest funding programmes in terms of health research: the Health theme of the Cooperation programme and the European Research Council (ERC). These programmes continue to exist under the Horizon2020 program.

Other funding organizations, such as the U.S. National Institutes of Health (NIH), also consisted of multiple sub-organizations or -programmes. These sub-organizations or –programmes were not denoted separately because their funding mechanisms varied less. Their funding mechanisms were well-captured by providing one overarching description for the funding mechanism of the overarching organization (the U.S. NIH).

### Excluded funding organizations

This study excluded several types of funding organizations:

Product development partnerships (PDPs) and other public private partnerships (PPPs) can also be funders of health research, but are funded themselves by governments, philanthropies and the for-profit sector. Because they are intermediate funding organizations, we did not include them in this study. Another reason for this was that most PDP’s have narrowly defined remits and are therefore of less interest with regards to priority setting processes (PPPs often have broader remits). We note that the annual funds received by nine PDP’s in the area of neglected diseases (largest is PATH, $ 81 million) [17] and the annual expenditures of the PPP that reports itself to be the world’s largest in the life sciences (the Innovative Medicines Initiative (IMI), $ 455 million) [18] were lower than the annual expenditures of the ten largest public and philanthropic funders reported in this study.

Private for-profit funding was also not included in our analysis. We only aimed to map public and philanthropic funders (private for-profit health research funders are mapped elsewhere [19]).

We excluded single disease funders. Although the majority of philanthropic funders of health research focuses on one disease,[20] the largest philanthropic funders of health research tend to fund across multiple disease areas (with some exceptions [21, 22]). Because they focus on more than one area of health, funders with more expansive portfolios are also of more interest with regards to priority setting processes. (We note that the expenditures of the largest single disease funder that we found, Cancer Research UK, was lower than the annual expenditures of the top-10 funders in this study ($ 548.7 million)).

Finally, many funders were both funders and organizations that conduct research themselves. When funding organizations funded research intramurally, this was most often a small percentage of their funding. Some funding organizations, however, funded research almost exclusively intramurally. The inclusion of these organizations as funders (as opposed to them being considered research institutes) depended on several aspects. We included such organizations: if they clearly were the primary public funder of health research of a country; if they were a philanthropic funder with a private research fund; or if they were clearly a funding organization for other reasons, such as when their employees worked at a great number of different universities or health research institutes throughout the country. We also took notice of whether the organization was denoted as a funder in other publications and reports. If the organization was considered to be primarily a research institute, and not a funder of health research, it was excluded.

### Assessing health research expenditures

For all the funding organizations that followed from our search, publicly available numbers on the organizations’ annual health research expenditures were sought via annual reports or websites. When we were not able to find data on annual expenditures in the public domain, we contacted funders to ask if they could provide us with their annual expenditures on health research.

Research funders differ in terms of how they report annual health research expenditures (i.e., actual expenditures, commitments or budgets); in terms of what the expenditures cover (i.e., total expenditures on health research excluding operational costs (for managing the funding organization), total expenditures including also operational costs, or total overall turnover for the funder over a single fiscal year (the latter was only collected if the funding organization exclusively funded health research)); and in terms of the research areas that their reported numbers pertain to (i.e., only health research, health and biological research, or life sciences research). For each funder we extracted data on annual health research expenditures in a step-wise manner, reporting always the actual expenditures excluding operational costs in the area of health research when possible. When these numbers were not available, we reported the next best available number, following the order in the categories provided above. Data were extracted for the most recent year available. Unfortunately, the data available on most funders was limited to one of the categories listed above, making it impossible for us to analyse the impact of these different formats for reporting funding data. We note that the data from the funders in the top-10 all relate only to health research, all concern actual expenditures or commitments, and for all except one all exclude operational costs.

Finally, organizations’ expenditures were made comparable. To do so, we firstly deflated organizations’ expenditures in the national currency to the year 2013 using Gross Domestic Product (GDP) deflators from the International Monetary Fund (IMF) World Economic Outlook Database of April 2014 (when they were not available for the year 2013). Secondly, we converted the inflation-corrected expenditures to US dollars using the World Bank Official exchange rates for the year 2013. As a secondary outcome, we calculated funding organizations’ health research expenditures as 2013 purchasing power parity-adjusted (PPP) US dollars (these are not reported in this article, but are available on [www.healthresearchfunders.org](http://www.healthresearchfunders.org) ).[23, 24]

## Assessing the funding patterns and mechanisms of the ten largest funders of health research

After the ten largest funding organizations of health research were identified, data were collected on their funding patterns and mechanisms. For each organization, data were collected on two aspects of the funder’s funding patterns and mechanisms: 1) the funding scheme(s) used to distribute funding; 2) the amount of funding allocated to a list of 20 health problems from the Global Burden of Disease (GBD) classification.[25]

When we included overarching funding organizations and their sub-organizations or -programmes, we assessed all organizations for their funding patterns and mechanisms. Therefore, for that analysis we collected data on 13 funding (sub-)organizations in total.

All data were collected from online reporting databases, annual reports, official websites, or other information sources. After this, each funder was invited to participate in an interview. Before the interview, a document with collected data was made available to a representative of the funder. Before and during the interviews, representatives were asked to add, adjust or confirm data.

Here below, we provide details on the aspects of funders’ funding patterns and mechanisms that data were collected on.

### 1. Funding schemes used to distribute funding

Data were collected about how much funding was distributed by the funders via various funding schemes (e.g. intramural vs. extramural research; or project grants vs. funding to organizations vs. grants that focus more on the excellence of researchers than of the proposed project (‘people grants’) vs. other funding). The manner in which funders reported on these funding schemes differed, so a generic framework was developed to report this in a comparable manner across funders (see Table 4 in the main text of this article), which was developed on the basis of the categorization systems used by funders included in our analysis.

To populate the framework we first assessed how much funding was allocated by each funder intramurally and extramurally. Whenever publicly available documents provided information on the funding organization’s operational expenditures separate from the organization’s research expenditures, we did not include funding for operational expenditures, but only funding actually intended for research (i.e., funding for research projects, including project grants, ‘people grants’, programme grants, funding distributed to organizations, and other research expenditures). We excluded expenditures for research education and training, but because there can be an overlap between these activities and research activities, particularly in the case of post-doctoral fellowships, we did collect expenditures toward research education and training separately.

Second, for all extramural funding, we assessed how much was allocated via project grants, via ‘people grants’ that place more importance on the individual excellence of the researcher than on the quality of a proposed project, via organizational funding, and via other research funding distribution mechanisms, including funding for communication support (e.g. funding for knowledge translation activities).

Third, within research project grants, we further collected data on how much funding was distributed using:

- Untargeted funding schemes: The funding calls under these schemes were not targeted toward prioritized research areas, topics or questions and research proposals in all areas were welcome (there were also described by funders as “unsolicited” or “response-mode” approaches).
- Untargeted funding schemes, earmarked for broad areas that are inclusive of all health research: All areas of health research were funded under these funding schemes, so researchers from all areas could apply, but there were pre-determined amounts of funding available for various broad areas of health research (an example of a funder who funds this way is the U.S. NIH which distributes its funding largely via its more specialized, topic-specific institutes).
- Targeted funding schemes: These funding schemes issued calls for prioritized areas or topics of health research (there were also described by funders as “solicited” or “strategic” approaches to funding distribution).
- Highly targeted funding schemes: These funding schemes came in the form of research contracts, tenders or prizes.

We did not collect data on additional funding distribution mechanisms such as the use of grants to small businesses (when reported this was categorized under project funding) and funding for PDPs and PPPs (when reported this was categorized under organizational funding), because these data were difficult to collect and compare for all funders.

Fourth, based on funders’ expenditures via various schemes, we classified funders ‘main funding distribution mechanism’ in two ways. First, as:

- Intramural
  - Fully (100% intramural)
  - Largely (>65% intramural)
- Extramural
  - Fully (100% extramural)
  - Largely (>65% extramural)
- Mixed (35-65% extramural or intramural)

Second, as:

- Untargeted
  - Fully (untargeted project funding + ‘people grants’ = 100%)
  - Largely (untargeted project funding + ‘people grants’ > 50%)
- Untargeted, with earmarked funding for broad areas that are inclusive of all health research
  - Fully (untargeted earmarked project funding = 100%)
  - Largely (untargeted earmarked project funding > 50%)
- Targeted, issuing calls under prioritized health research areas or topics
  - Fully (targeted funding = 100%)
  - Largely (targeted funding > 50%)
- Highly targeted, in the form of research contracts, tenders or prizes
  - Fully (highly targeted funding = 100%)
  - Largely (highly targeted funding > 50%)
- Organizational funding
  - Fully (organizational funding = 100%)
  - Largely (organizational funding > 50%)

Mixed approachesThis analysis took some generalizations, because several funders combined various funding schemes, in which case we described the most prominent modes of funding (i.e. the scheme through which the most funding was allocated). When we included overarching funding organizations and their sub-organizations or -programmes, we assessed only the sub-organizations and programmes (i.e., the CDMRP, the Health theme of the EC FP7 Cooperation programme and the ERC, but not the US DoD or the EC as a whole). Therefore, for we classified 11 funding (sub-)organizations to the abovementioned categories.

There were no funders with whom the majority of funding consisted of highly targeted funding (e.g. research contracts, tenders and prizes) or who provided more than 50% of their funding through organizational funding, although there are funding organizations that fund in this manner, such as the Global Health Investment Fund (GHIF) [26] and the UK National Institute for Health Research (NIHR) respectively.[27]

### 2. Funding patterns towards diseases

Data were collected about how much funding for health research was allocated by funders towards 20 causes of burden of disease. The health problems and sub-categories of health problems that had the largest burden of disease within their category were chosen, under the assumption that if data were not available for these health problems, they would likely also not be available for health problems with smaller burdens.[25]

In principle, we only denoted funding data that were developed using an indexing system. For one funder (the Health theme of the EC FP7 Cooperation programme), at their own request, we compiled data on the basis of the target areas of their funding programmes. We note that such reporting is less accurate than reporting based on indexing (e.g., if a funder has a specific funding program for cardiovascular disease, it might also fund research on cardiovascular disease outside that program, which will not be included using this method).

Finally, it was denoted if funders used a classification system to report their funding data. Most funding classification systems choose to classify research along two axes: health areas and research type or purpose.[28] WHO’s recommended framework for monitoring health research funding also recommends to collect both.[29] Therefore, we noted if a classification system was used to classify funding to various health areas and we assessed if organizations presented statistics about the allocation of funding to various research types (e.g. biomedical research, clinical research, epidemiological research or health systems research [30]). We also denoted whether funders presented statistics on which countries or regions were recipients of their funding.

## References

1. Røttingen J-A, Regmi S, Eide M, Young AJ, Viergever RF, Årdal C, Guzman J, Edwards D, Matlin SA, Terry RF: **Mapping available health R&D data: what’s there, what’s missing and what role for a Global Observatory**. *Lancet* 2013, **382**:1286–1307.

2. **Health Research Web (HRWeb)** [https://www.healthresearchweb.org/]

3. Nason E: *Health and Medical Research in Canada: Observatory on Health Research Systems*. Cambridge: RAND Europe; 2008.

4. **Erawatch: Platform on Research and Innovation policies and systems** [http://erawatch.jrc.ec.europa.eu/]

5. **Table #301. Federal Obligations for Health Research and Development by Federal Agency.** [http://report.nih.gov/DisplayRePORT.aspx?rid=579]

6. Tediosi F, Compagni A, Vuolo E: *Analisi Del Sistema Di Finanziamento Della Ricerca Sanitaria in Italia*. Centro di Ricerche sulla Gestione dell’Assistenza Sanitaria e Sociale (CERGAS), dell’Università Commerciale Luigi Bocconi; 2010.

7. Watt A: *Health and Medical Research in Australia: Observatory on Health Research Systems*. Cambridge: RAND Europe; 2008.

8. Hassan E: *Health and Medical Research in France: Observatory on Health Research Systems*. Cambridge: RAND Europe; 2009.

9. Burgdorf JR: *Health and Medical Research in Japan: Health Research Observatory*. Cambridge: RAND Europe; 2008.

10. Scoggins B: *Health and Medical Research in New Zealand: Health Research Observatory*. Cambridge: RAND Europe; 2008.

11. Marjanovic S, Chonaill SN: *Health and Medical Research in Singapore: Observatory on Health Research Systems*. Cambridge: RAND Europe; 2010.

12. Tiessen J: *Health and Medical Research in Sweden: Observatory on Health Research Systems*. Cambridge: RAND Europe; 2008.

13. Archontakis F: *Health and Medical Research in Spain: Health Research Observatory*. Cambridge: RAND Europe; 2008.

14. Hargreaves S: *Health and Medical Research in the United Kingdom: Observatory on Health Research Systems*. Cambridge: RAND Europe; 2008.

15. Shergold M: *Health and Medical Research in the United States: Observatory on Health Research Systems*. Cambridge: RAND Europe; 2008.

16. *Financing Global Health 2012: The End of the Golden Age?* Seattle: Institute for Health Metrics and Evaluation; 2013.

17. Moran M, Guzman J, Henderson K, Liyanage R, Wu L, Chin E, Chapman N, Abela-Oversteegen L, Gouglas D, Kwong D: *G-FINDER 2012 – Neglected Disease Research & Development: A Five Year Review*. Sydney: Policy Cures; 2012.

18. **The Innovative Medicines Initiative: Introducing IMI: The Budget** [http://www.imi.europa.eu/content/mission]

19. **The EU Industrial R&D Investment Scoreboard** [http://iri.jrc.ec.europa.eu/scoreboard.html]

20. Myers ER, Alciati MH, Ahlport KN, Sung NS: **Similarities and differences in philanthropic and federal support for medical research in the United States: an analysis of funding by nonprofits in 2006-2008.** *Acad Med* 2012, **87**:1574–81.

21. *Saving Lives through Research: Annual Report and Accounts 2012/13*. Cancer Research UK; .

22. *Research Facts 2012-13*. American Heart Association; 2014.

23. Young AJ, Terry RF, Røttingen J-A, Viergever RF: **Global biomedical R&D expenditures.** *N Engl J Med* 2014, **370**:2451.

24. Young AJ, Terry RF, Røttingen J-A, Viergever RF: **Global trends in health research and development R&D expenditures – the challenge of making reliable estimates for international comparison**. *Heal Res policy Syst* 2015, **13**:7.

25. **World Health Organization: Global Health Estimates (GHE)** [http://www.who.int/healthinfo/global_burden_disease/en/]

26. **Global Health Investment Fund** [http://ghif.com/]

27. *National Institute for Health Research: Annual Report 2012/2013*. .

28. Terry RF, Allen L, Gardner C, Guzman J, Moran M, Viergever RF: **Mapping global health research investments, time for new thinking - A Babel Fish for research data**. *Heal Res policy Syst* 2012, **10**:28.

29. **Research policy, HRSA indicators: Financing function** [http://www.who.int/rpc/health_research/concepts/indicators/en/index1.html]

30. Frenk J: **The new public health.** *Annu Rev Public Health* 1993, **14**:469–90.
