# Supplementary material for: The 10 largest public and philanthropic funders of health research in the world: what they fund and how they distribute their funds
Source: Health Res Policy Syst. 2016 Feb 18;14:12. doi: 10.1186/s12961-015-0074-z (PMC4759950; doi:10.1186/s12961-015-0074-z)
Supplement: Additional file 2: — Sources for identification of health research funding organizations. (DOCX 45 kb) [file 12961_2015_74_MOESM2_ESM.docx]

# Additional File 2. Sources for identification of health research funding organizations

To aid future analyses like the one conducted for this article, we provide here an overview of various sources that helped us identify the main public and philanthropic funders of health research globally and that that resulted in the 55 organizations listed in the main text of this article.

For *public* funders, we found articles,[1] reports,[2] and online lists and databases, such as the Health Research Web,[3] Erawatch,[4] World Report,[5] and other smaller lists of members of forums or other collaborative groups,[6] that presented some information on public funders of health research. However, these resources were not complete, and for many funders these resources did not report annual health research expenditures. In addition, for several countries, mainly high-income nations, country-based reports were available outlining the country’s health research system [3, 4, 7–17] or its funding [18, 19]. We found no overall list of all public health research funders and their annual health research expenditures. Lastly, we note that estimates of public support for health research by various organizations can vary depending on the method of calculation.[18, 20, 21]

For *philanthropic* funders we found no international lists of philanthropic funders of health research, although for the European Union, the EUFORI study was collecting data on philanthropic funders of research, including health research, at the time of this study, but their results were not yet available.[22] For individual countries, such lists were available. For the UK, for example, a list was available through the Association of Medical Research Charities, which included data on the organizations’ annual health research expenditures.[23] For the US, we found several databases of charities and foundations. We were able to obtain a list of health research funders, including annual expenditures on health research, from the Foundation Center.[24] However, the foundation centre list was not exhaustive because public charities are excluded from the Foundation Center database. Key American funders of health research such as the American Cancer Society and the American Heart Association are not included in its database.[25] Moreover, we found the data reported by the Foundation Center on organizations’ health research spending, such as the Bill and Melinda Gates Foundation, to be highly variable and different from other sources of information on those same funders,[2] which made us doubt the reliability of these data. We found another list by the National Center for Charitable Statistics (NCCS)*,* a U.S. based organization that tracks data on non-profit organizations and their activities for use in research. This list did include charities, but it did not have data of heath research expenditures of foundations, only of organizations’ total assets and receipts, (based on the National Taxonomy of Exempt Entities (NTEE) classification).[26] There are several other NTEE-based lists like that of the NCCS available. Finally, we found a report on charitable funders of health research in the US, based on the member organizations of the Health Research Alliance, but the article does not present a list of individual charities’ expenditures on health research.[27]

We note that estimates of philanthropic support for health research in the US vary greatly, from less than 1 billion USD at one source,[20] to more than 5 billion at another,[28] showing the necessity for an inclusive, accurate, publicly accessible list of philanthropic funders, including their health research expenditures. For other regions, we were not aware of databases of philanthropic funders of health research.

# References

1. Sun GH, Steinberg JD, Jagsi R: **The calculus of national medical research policy--the United States versus Asia.** *N Engl J Med* 2012, **367**:687–90.

2. Moran M, Guzman J, Henderson K, Liyanage R, Wu L, Chin E, Chapman N, Abela-Oversteegen L, Gouglas D, Kwong D: *G-FINDER 2012 – Neglected Disease Research & Development: A Five Year Review*. Sydney: Policy Cures; 2012.

3. **Health Research Web (HRWeb)** [https://www.healthresearchweb.org/]

4. **Erawatch: Platform on Research and Innovation policies and systems** [http://erawatch.jrc.ec.europa.eu/]

5. Collins F, Beaudet A, Draghia-Akli R, Gruss P, Savill J, Syrota A, Dautry A, Ulfendahl M, Walport M, Onken J, Glass RI: **A database on global health research in Africa**. *Lancet Glob Heal* 2013, **1**:e64–e65.

6. **International Stem Cell Forum** [http://www.stem-cell-forum.net/]

7. Nason E: *Health and Medical Research in Canada: Observatory on Health Research Systems*. Cambridge: RAND Europe; 2008.

8. Tediosi F, Compagni A, Vuolo E: *Analisi Del Sistema Di Finanziamento Della Ricerca Sanitaria in Italia*. Centro di Ricerche sulla Gestione dell’Assistenza Sanitaria e Sociale (CERGAS), dell’Università Commerciale Luigi Bocconi; 2010.

9. Watt A: *Health and Medical Research in Australia: Observatory on Health Research Systems*. Cambridge: RAND Europe; 2008.

10. Hassan E: *Health and Medical Research in France: Observatory on Health Research Systems*. Cambridge: RAND Europe; 2009.

11. Burgdorf JR: *Health and Medical Research in Japan: Health Research Observatory*. Cambridge: RAND Europe; 2008.

12. Scoggins B: *Health and Medical Research in New Zealand: Health Research Observatory*. Cambridge: RAND Europe; 2008.

13. Marjanovic S, Chonaill SN: *Health and Medical Research in Singapore: Observatory on Health Research Systems*. Cambridge: RAND Europe; 2010.

14. Tiessen J: *Health and Medical Research in Sweden: Observatory on Health Research Systems*. Cambridge: RAND Europe; 2008.

15. Archontakis F: *Health and Medical Research in Spain: Health Research Observatory*. Cambridge: RAND Europe; 2008.

16. Hargreaves S: *Health and Medical Research in the United Kingdom: Observatory on Health Research Systems*. Cambridge: RAND Europe; 2008.

17. Shergold M: *Health and Medical Research in the United States: Observatory on Health Research Systems*. Cambridge: RAND Europe; 2008.

18. **Table #301. Federal Obligations for Health Research and Development by Federal Agency.** [http://report.nih.gov/DisplayRePORT.aspx?rid=579]

19. *UK Health Research Analysis 2009/2010*. London: UK Clinical Research Collaboration (UKCRC); 2010.

20. *Truth and Consequences: Health R&D Spending in The U.S. (FY11-12)*. Alexandria: Research!America; .

21. Young AJ, Terry RF, Røttingen J-A, Viergever RF: **Global biomedical R&D expenditures.** *N Engl J Med* 2014, **370**:2451.

22. **EUFORI study** [http://euforistudy.eu/]

23. **Association of Medical Research Charities (AMRC): Member list** [http://www.amrc.org.uk/our-members/member-directory]

24. **The Foundation Center** [http://foundationcenter.org/]

25. *Research Facts 2012-13*. American Heart Association; 2014.

26. **National Center for Charitable Statistics (NCCS)** [http://nccs.urban.org/]

27. Myers ER, Alciati MH, Ahlport KN, Sung NS: **Similarities and differences in philanthropic and federal support for medical research in the United States: an analysis of funding by nonprofits in 2006-2008.** *Acad Med* 2012, **87**:1574–81.

28. Wadman M: **Biomedical philanthropy: state of the donation.** *Nature* 2007, **447**:248–50.
